# Cavernome portal chez l’enfant révélé par une hémorragie digestive: à propos d’un cas

**DOI:** 10.11604/pamj.2016.25.158.10616

**Published:** 2016-11-14

**Authors:** Idrissa Basse, Ndéye Rama Diagne Guèye, Dina Cyrienne Obambi Diop, Ndiémé Ndiaye Diawara, Aïssatou Ba, Ndiogou Seck, Aliou Thiongane, Abou Ba, Aliou Abdoulaye Ndongo, Amadou Lamine Fall, Djibril Boiro, Lamine Thiam, Marie Mbengue

**Affiliations:** 1 Hôpital pour Enfants de Diamniadio, Université de Thiès, Thiès, Sénégal; 2 Hôpital d’Enfants Albert Royer, Université Cheikh Anta Diop de Dakar, Dakar, Sénégal; 3 Hôpital Régional de Saint-Louis, Service de Pédiatrie, Université de Saint-Louis, Saint-Louis, Sénégal; 4 Hôpital Abass Ndao, Service de Pédiatrie, Université Cheikh Anta Diop de Dakar, Dakar, Sénégal; 5 Hôpital de la Paix de Ziguinchor, Université de Ziguinchor, Ziguinchor, Sénégal

**Keywords:** Cavernome portal, échographie abdominale, endoscopie, scanner abdominal, enfant, Portal cavernoma, abdominal ultrasound, endoscopy, abdominal CT scan, child

## Abstract

Le cavernome portal est une anomalie vasculaire veineuse caractérisée par la formation d’un réseau de veines dont le calibre est augmenté et au sein duquel chemine un sang portal. Il est la conséquence d’une occlusion thrombotique et toujours chronique du système porte extra hépatique. C’est une des causes les plus fréquentes d’hypertension portale chez l’enfant. Ainsi sa gravité est surtout liée au risque important d’hémorragies digestives. Très peu de cas ont été décrits dans la littérature notamment africaine. Nous rapportons l’observation d’un garçon de 4 ans reçu pour hématémèse de grande abondance, méléna et vertiges qui présentait à l’examen un syndrome anémique. Le bilan biologique retrouvait une anémie sévère hypochrome microcytaire avec une fonction rénale ethépatique normale. L’endoscopie oeso-gastrique montrait des varices oesophagiennes grade III avec signes rouges. L’échographie abdominale mit en évidence un lacis veineux portal en faveur d’un cavernome. Le scanner abdominal confirmait le cavernome porte avec syndrome d’hypertension portale et anomalie vasculaire à type d’abouchement ectopique de la veine splénique au tronc formé par la veine gonadique et la veine mésentérique inférieure. Sur le plan thérapeutique une transfusion sanguine a été effectuée et il a été mis sous bétabloquant. Le cavernome portal peut être une complication majeure de malformations vasculaires souvent méconnues. Il faut y penser devant toute hémorragie digestive chez l’enfant. La prise en charge doit être urgente et adaptée pour éviter une évolution fatale.

## Abstract (en)

Portal cavernoma is a venous vascular anomaly characterized by the formation of a network of veins whose caliber is increased and carrying portal blood. It is due to a thrombotic and always chronic occlusion of the extra-hepatic portal venous system. This is one of the most common causes of portal hypertension in children. Its severity is mainly associated with an high risk of gastrointestinal haemorrhage. Very few cases have been described mainly in African literature. We report the case of a 4-year old boy admitted with very abundant haematemesis, melena and dizziness associated with anemic syndrome on examination. Laboratory tests showed severe microcytic hypochromic anemia with normal renal and hepatic function. Gastrointestinal endoscopy showed esophageal varices (grade III) with red signs. Abdominal ultrasound showed portal vein formation resulting in the classic “spiderweb”, in favor of a cavernoma. Abdominal CT scan confirmed portal cavernoma associated with portal hypertensive syndrome and vascular anomaly like an ectopic splenic vein anastomosis with the trunk formed by the gonadal vein and the inferior mesenteric vein. Therapeutic approach was based on blood transfusion and beta-blocker treatment. Portal cavernoma can be a major complication of vascular malformations often unknown. In case of gastrointestinal haemorrhage in children, diagnosis should be suspected. Its management requires early treatment and should be adapted to the patient’s condition in order to prevent a fatal evolution.

## Introduction

Un cavernome portal est un réseau formé de veines dont le calibre, initialement millimétrique ou microscopique, est augmenté de diamètre et au sein desquelles chemine un sang portal hépatopète. C’est l’aboutissement d’un mécanisme de compensation naturelle qui se met en œuvre dès que la veine porte s’obstrue. Il est la conséquence d’une occlusion, thrombotique et toujours chronique, du système porte extra-hépatique [1]. Chez l'enfant, le cavernome portal est une cause majeure d'hypertension portale dite «pré ou infra-hépatique» ou encore «extra-hépatique». Le diagnostic repose essentiellement par l’imagerie médicale [2]. Très peu de cas ont été rapportés dans la littérature africaine notamment en Afrique Sub-saharienne. Nous rapportons un cas suivi à l’Hôpital pour Enfants de Diamniadio (30km de Dakar).

## Patient et observation

**Figure 1:**
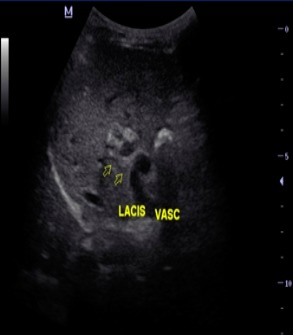
Image échographique montrant le lacis veineux portal

**Figure 2:**
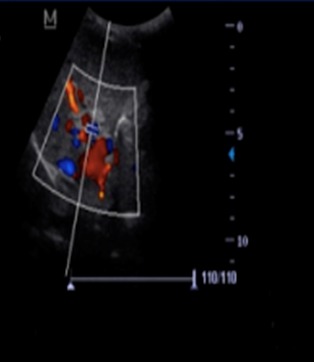
Image échographique montrant le lacis veineux portal avec flux sanguin doppler

**Figure 3:**
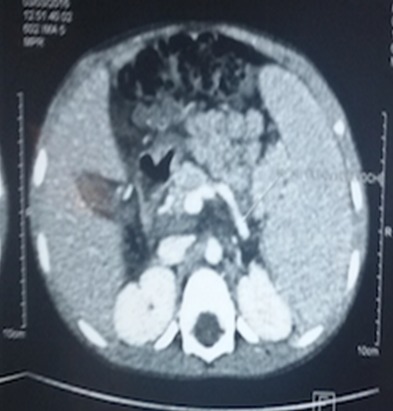
Scanner abdominal montrant le cavernome porte

**Figure 4:**
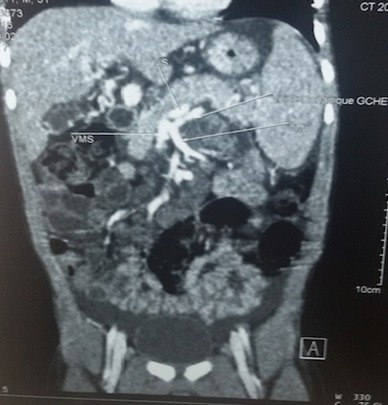
Scanner abdominal montrant la malformation vasculaire

O.M est un garçon âgé de 4 ans qui n’a eu aucun antécédent pathologique médical ni chirurgical particulier, sans notion d’hospitalisation durant la période néonatale avec un bon développement psychomoteur et un statut vaccinal complet selon le programme élargi de vaccination du Sénégal. Il est le 4^ème^ d’une fratrie de 5 enfants, ses frères et sœurs sont vivants et bien portants. Il a été reçu dans notre service pour hématémèse de grande abondance, méléna et vertiges chez qui l’examen notait un syndrome anémique et un syndrome hémorragique avec un doigtier souillé de selles noirâtres au toucher rectal. Par ailleurs son poids et sa taille étaient normaux. Le bilan biologique retrouvait une anémie sévère avec un taux d’hémoglobine à 5,4g/dL hypochromemicrocytaire, un taux de plaquettes normal, les fonctions rénales et hépatiques étaient normales, l’AgHbs était négatif. L’endoscopie OGD montrait des varices oesophagiennes grade III avec signes rouges. L’échographie abdominale mit en évidence une vésicule biliaire hypotrophique avec une paroi fibreuse et un lacis veineux portal en faveur d’un cavernome (Figure 1 et Figure 2). Le scanner abdominal confirmait le cavernome porte associé à un syndrome d’hypertension portale et une anomalie vasculaire à type d’abouchement ectopique de la veine splénique au tronc formé par la veine gonadique et la veine mésentérique inférieure (Figure 3 et Figure 4). Sur le plan thérapeutique une transfusion sanguine a été effectuée et il a été mis sous bétabloquant. Cependant la ligature des varices œsophagiennes prévue n’a pu être effectuée car au troisième jour de son hospitalisation l’enfant décédera d’une hémorragie digestive haute et basse cataclysmique.

## Discussion

La première description du cavernome portal a été faite en 1903 lors de l'autopsie d'un patient de 44 ans décédé d'une thrombose veineuse mésentérique extensive, au terme de huit années de douleurs abdominales chroniques inexpliquées [3]. Elle est la cause la plus fréquented'hypertension portale chez l'enfant, il peut être secondaire ou idiopathique. Sa gravité est majeure en raison de la fréquence et de la sévérité des hémorragies digestives secondaires à la rupture des varices œsophagiennes(VO) [4]. Aux Etats Unis l’incidence est estimée à 1% dans la population générale [5]. Dans nos régions très peu de cas ont été rapportés. Une étude menée en Tunisie en 2001 ne décrivait que 19 observations sur une période de 25 ans, une autre faite au Maroc rapportait 11 cas de 2003 à 2012 [2, 4]. Ailleurs des cas isolés de cavernome porte de l’enfant ont été décrits par Ba A. et al. au Sénégal ainsi que Ben Chaabane N. et al. en Tunisie avec des modes de révélations variables mais qui restent toujours dominées par les hémorragies digestives [6, 7]. Le diagnostic se fait le plus souvent durant l’enfance comme en témoigne l’âge de notre patient (4ans) ainsi que l’âge moyen des patients dans la série de Hachicha M. et al. qui est de 6 ans avec cependant des extrêmes de 1 mois et 12ans; Tadmori I. et al. Rapportaient une série de 11 cas avec un âge moyen de 8ans et des extrêmes de 2 et 15 ans. On note également une prédominance masculine dans la plupart des séries et des cas rapportés dans la littérature.

En dehors des hémorragies digestives qui en font toute la gravité, d’autres manifestations cliniques que nous n’avons pas objectivé chez notre patient telles que la splénomégalie, les douleurs abdominales ainsi que le retard de croissance sont fréquentes [1, 6]. Sur le plan biologique notre patient avait un taux de prothrombine bas. En effet l'apparition d'une thrombose portale est le plus souvent la conjonction d'une cause locale et d'une maladie prothrombotique qui doivent être systématiquement recherchées. Ces anomalies seraient secondaires à une surconsommation par un mécanisme de coagulation intravasculaire disséminée au sein des capillaires formant le cavernome [1, 8]. L’endoscopie retrouvait des varices œsophagiennes; ces varices sont fréquentes, 90 à 95% des cas [1]. Les causes du cavernome sont celles de la thrombose portale à l'exception des malformations vasculaires mais chez l'enfant il s'agit le plus souvent de traumatismes directs de la veine porte aux premiers rangs desquels l'on trouve le cathétérisme de la veine ombilicale [9]. Par contre pour notre patient on ne retrouvait aucune notion de cathétérisme ombilical mais le scanner abdominale objectivait une anomalie vasculaire à type d’abouchement ectopique de la veine splénique et du tronc formé par la veine gonadique et la veine mésentérique inferieure laquelle malformation nous apparaissait être un facteur favorisant clé dans la survenue du cavernome. L'indication thérapeutique pose souvent des difficultés pour le praticien et repose surtout sur le traitement symptomatique: bêta-bloquant, sclérothérapie et/ou ligature élastique des VO et dérivation porto-systémique [4]. Notre patient a été mis sous bêta-bloquant ce qui n’a pu empêcher la récidive d’une hémorragie digestive massive à l’issue dramatique. Dans la série rapportée par Hachicha M. et al. près de 50% des cas ont eu une issue favorable avec l’association bêta-bloquant, sclérothérapie et/ou chirurgie. De meilleurs résultats ont été notés dans l’étude menée par Tadmori I. et al. au Maroc où sous bêta-bloquant seul pour 7 cas, aucun cas de décès n’a été noté.

## Conclusion

Le cavernome portal est une cause majeure de l'hypertension portale en pédiatrie. Son diagnostic est fait par l’imagerie et exige une prise en charge multidisciplinaire de haut niveau entre pédiatres, radiologues et chirurgiens infantiles, permettant de prendre une décision précoce et adaptée qui ne se réalisera qu’à travers une maîtrise des différents outils thérapeutiques. Le pronostic dépend de la qualité de cette prise en charge qui pose souvent problème dans nos pays en développement.
